# Structural Designing of a MEMS Capacitive Accelerometer for Low Temperature Coefficient and High Linearity

**DOI:** 10.3390/s18020643

**Published:** 2018-02-22

**Authors:** Jiangbo He, Wu Zhou, Huijun Yu, Xiaoping He, Peng Peng

**Affiliations:** 1 School of Mechanical Engineering, Xihua University, Chengdu 610039, China; 2 School of Mechanical and Electrical Engineering, University of Electronic Science and Technology of China, Chengdu 611731, China; yuhjuestc@126.com (H.Y.); ppuestc@163.com (P.P.); 3 Institute of Electronic Engineering, China Academy of Engineering Physics, Mianyang 621900, China; hexpiee@263.net

**Keywords:** MEMS, capacitive accelerometer, temperature coefficient, linearity error

## Abstract

The low temperature coefficient and high linearity of the input-output characteristics are both required for high-performance microelectromechanical systems (MEMS) capacitive accelerometers. In this work, a structural designing of a bulk MEMS capacitive accelerometer is developed for both low temperature coefficient and high linearity. Firstly, the contrary effect of the wide-narrow gaps ratio (WNGR) on the temperature coefficient of the scale factor (TCSF) and linearity error is discussed. Secondly, the ability of an improved structure that can avoid the contrary effect is illustrated. The improved structure is proposed in our previous work for reducing the temperature coefficient of bias (TCB) and TCSF. Within the improved structure, both the TCSF and linearity error decrease with increasing WNGR. Then, the precise designing of the improved structure is developed for achieving lower TCB, TCSF, and linearity error. Finally, the precise structural designing is experimentally verified.

## 1. Introduction

High-precision MEMS capacitive accelerometers are increasingly needed in numerous applications including inertial navigation [1], gravity measurement [2], vibration measurements [3], and so on. The low temperature coefficient and high linearity of the input-output characteristics are both required for the high-performance MEMS capacitive accelerometers, especially for those applied in inertial navigation [4,5]. Temperature coefficient can be induced by the thermal stress and temperature dependence of material properties [6,7]. The thermal stress is induced by the mismatch of thermal expansion among device layer, substrate, and package [8]. Linearity error (or nonlinearity) is the inherent drawback of capacitive accelerometers because the gap variation is used to detect the acceleration [9]. Even if the closed-loop detection principle is employed, linearity error still exists due to uncertainties, such as manufacturing errors [10]. Although the temperature coefficient and linearity error can both be compensated actively [11,12], more complex circuitry is required. 

The temperature coefficient and linearity error are both related to the device dimensions. Thus, the careful designing of device dimensions is necessary for both low temperature coefficient and linearity error. In our previous work [6], the temperature coefficient of the bias (TCB) and temperature coefficient of the scale factor (TCSF) are studied in detail. An improved structure is also designed for both low TCB and TCSF (TCB < 1.03 mg/°C and TCSF < 66 ppm/°C). However, TCB and TCSF are still high for high-performance accelerometers. For example, TCB is much higher than the results in several literatures (100 μg/°C in [13] and [14], 200 μg/°C in [4] and [15], 300 μg/°C in [16]). As such, more improvement of design is required to achieve lower TCB and TCSF. In addition, the linearity is not considered in the previous work. In this work, the temperature coefficient and linearity error are designed simultaneously. Firstly, the contrary effect of the wide-narrow gaps ratio (WNGR) on TCSF and linearity error is discussed. Then, a precise design to improve the structure is developed for both low temperature coefficients and linearity error.

## 2. Fabrication and Detection Principle

**Figure 1:**
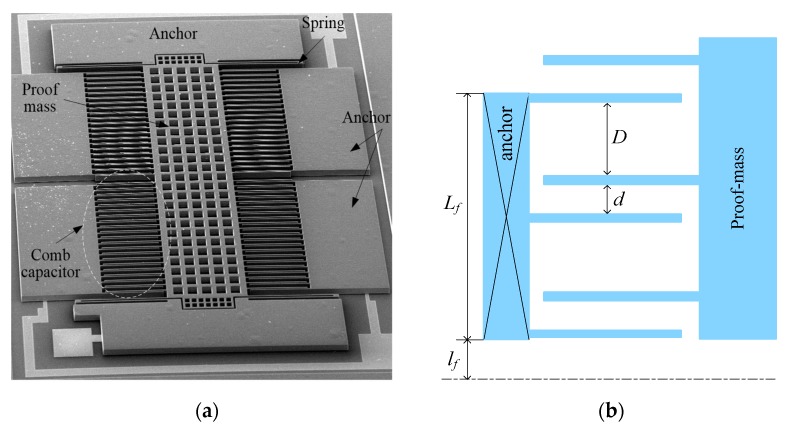
The structure of the MEMS accelerometer. (**a**) Scanning electron microscopy (SEM); (**b**) Scheme diagram of dimensions for a comb capacitor.

The structure of the MEMS capacitive accelerometer studied in this work is shown in Figure 1. Anchors and sensing elements (proof mass, comb capacitors, and springs) are made of single crystal silicon. The bottom substrate is made of Pyrex 7740 glass. The accelerometer is fabricated based on a bulk silicon process, which was detailed before in [7]. In short, the process begins with a Cr/Au metallization process on a Pyrex 7740 glass wafer. Then, boron doping by diffusion is performed on a (100) silicon wafer, and then DRIE is employed to define sensing elements and anchors. Next, the silicon wafer was flipped and bonded to the glass wafer by anodic bonding. Finally, the undoped silicon is completely dissolved to leave the sensing elements and anchors.

**Figure 2:**
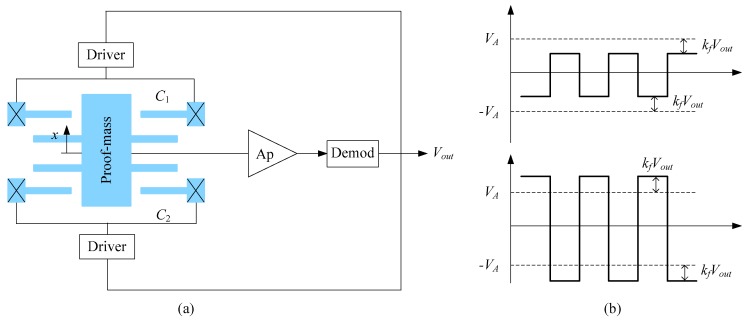
Scheme diagram of the self-balancing differential capacitive principle. (**a**) Schematic block diagram; (**b**) Graphs of waveforms on the fixed plate.

The capacitances of the accelerometer are detected by the self-balancing bridge principle, as depicted in Figure 2. The inertial force makes the proof mass move and is balanced by the spring force. The moving of the proof mass changes the capacitance of the accelerometer. Drivers provide AC signals to the fixed electrodes. The mov­able electrodes are connected to the output terminal through the reading circuitry. In response to a sensed acceleration, feed­back is provided from the output terminal to both drivers to adjust the amplitude of the AC signals on the fixed electrodes, as depicted in Figure 2b. The objective of the feedback is to null any AC signal on the movable electrodes. The detailed principle realizing the amplitude adjusting of the AC signals can be found in the literature [17]. In order to null any AC signal on the movable electrodes, the following equation must be satisfied

(1) $$C_{1}\left(V_{A}-\frac{1}{M}V_{out}\right)-C_{2}\left(V_{A}+\frac{1}{M}V_{out}\right)=0$$

where *V_A_* is the initial amplitude of the AC signal, *C*_1_ and *C*_2_ are the capacitance, *M* is a feedback coefficient depending on circuit parameters. Solving Equation (1) leads to

(2) $$V_{out}=M\frac{C_{1}-C_{2}}{C_{1}+C_{2}}V_{A}$$

## 3. Contrary Effect of the WNGR on TCSF and Linearity Error

### 3.1. Analytical Model for the Linearity Error

According to the electrostatic theory, capacitances of the accelerometer can be expressed as

(3) $$\begin{array}{l} C_{1}=\frac{2N\varepsilon \Omega }{d-x}+\frac{2\left(N-1\right)\varepsilon \Omega }{D+x}\\ C_{2}=\frac{2N\varepsilon \Omega }{d+x}+\frac{2\left(N-1\right)\varepsilon \Omega }{D-x} \end{array}$$

where *d* and *D* denote the narrow and wide gaps shown in Figure 1, respectively, *x* denotes the displacement of proof mass, *N* denotes the number of fixed electrodes in a comb capacitor, Ω represents the overlapping area in a pair of movable and fixed electrodes.

Substituting Equation (3) into (1) and expanding output *V_out_* using Taylor's theorem leads to

(4) $$V_{out}=\left(\frac{\left(\eta -1\right)x}{\eta d}+\frac{\left(\eta -1\right)x^{3}}{\eta^{2}d^{3}}\right)MV_{A}$$

where *η* denotes WNGR (the ratio of *D* to *d*). 

The displacement of proof mass caused by the input acceleration can be expressed as

(5) $$x=\frac{-ma}{K}$$

where *m*, *a*, and *K* denote the proof mass, acceleration, and the stiffness of springs, respectively. Substituting Equation (5) into (4), the output *V_out_* can be expressed as a function of the acceleration

(6) $$\begin{array}{l} V_{out}=k_{1}a+k_{3}a^{3}\\ k_{1}=-\frac{\eta -1}{\eta d}\frac{m}{K}MV_{A}\\ k_{3}=-\frac{\eta -1}{\eta^{2}d^{3}}{\left(\frac{m}{K}\right)}^{3}MV_{A} \end{array}$$

In Equation (6), *k*_1_ denotes the scale factor (or sensitivity), and *k*_3_ causes linearity error. According to the definition in [18] the linearity error represents the systematic deviation from the straight line that defines the nominal input-output relationship. The linearity error over full range can be expressed as

(7) $$\Delta_{non}=\frac{\max\left|V_{out}-V_{out\_linear}\right|}{V_{out\max}}\times 100$$

where *V_out_linear_* denotes the output obtained from the nominal input-output relationship. From Equation (6), it is known that “*k*_1_*a*” represents the nominal input-output relationship. Substituting Equation (6) into (7) leads to

(8) $$\Delta_{non}=\frac{k_{3}a_{\max}^{3}}{k_{1}a_{\max}+k_{3}a_{\max}^{3}}\approx \frac{k_{3}a_{\max}^{3}}{k_{1}a_{\max}}=\frac{1}{\eta }{\left(\frac{ma_{\max}}{Kd}\right)}^{2}$$

According to Equation (8), it is known that the linearity error decreases with the increasing of the narrow gap. However, increasing the narrow gap is against the detection due to the lower capacitance. Equation (8) also shows that higher WNGR results in lower linearity error, and even nulls the linearity error. 

In conclusion, high WNGR is the better way to achieve low linearity error. However, high WNGR may also influence the temperature coefficient. In next section, it is shown that WNGR has the contrary effect on TCSF and linearity error.

### 3.2. Illustration of the Contrary Effect

According to our previous work [6], TCSF of the MEMS accelerometer can be expressed as

(9) $$\mathrm{TCSF}=-\mathrm{TCS}+\left[-\alpha_{s}-\frac{\eta^{2}+1}{\eta^{2}-\eta }\left(\frac{l_{f}}{d}+\left(N-1\right)\left(\frac{\eta +1}{2}+\frac{w_{f}}{d}\right)\right)\left(\alpha_{eq}-\alpha_{s}\right)\right]$$

where *TCS* denotes the temperature coefficient of stiffness, *α_s_* denotes the coefficient of thermal expansion (CTE) of silicon, *α_eq_* is called as equivalent CTE, *N* denotes the fixed electrode number in a comb capacitor, and *l_f_* denotes the distance from the first fixed electrode to the midline, *w_f_* denotes the width of an electrode, as shown in Figure 1. TCS equals the sum of the temperature coefficient of elastic modulus and CTE. However, the temperature coefficient of elastic modulus is much higher than CTE [19]. Thus, TCS is mainly determined by the temperature coefficient of elastic modulus. Due to the high boron doped silicon, TCS is about 30 ppm/°C [6]. The equivalent CTE *α_eq_* represents the thermal deformation on the substrate top surface. Because the thermal stress enhances the thermal deformation on the substrate top surface, the equivalent CTE is higher than the CTE of Pyrex 7740 glass.

**Figure 3:**
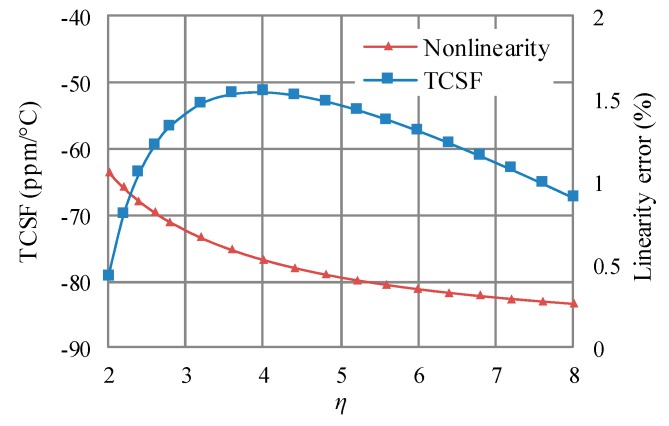
The effect of WNGR (*η*) on TCSF and linearity error.

From Equation (9), it is known that the relationship between TCSF and WNGR is nonlinear. The nonlinear relationship is shown in Figure 3. For comparison, the relationship between the linearity error and WNGR is also shown in Figure 3. Except for the equivalent CTE, the employed parameters (listed in Table 1) are the same as those in the previous work [6]. In this work, the minimum equivalent CTE is employed and equal to the CTE of the glass substrate (about 3.25 ppm/°C). The minimum equivalent CTE can be achieved by using the very soft adhesive for the die-attach [6]. From Figure 3, it can be seen that WNGR must be high for low linearity error. For example, a WNGR higher than 5.5 results in a linearity error lower than 0.5%. On the other side, TCSF achieves the minimum with the WNGR of 4. When WNGR is higher than 4, TCSF increases with increasing WNGR. Therefore, the effect of WNGR on TCSF and linearity error is contrary. 

### 3.3. Structural Designing for Avoiding the Contrary Effect

**Figure 4:**
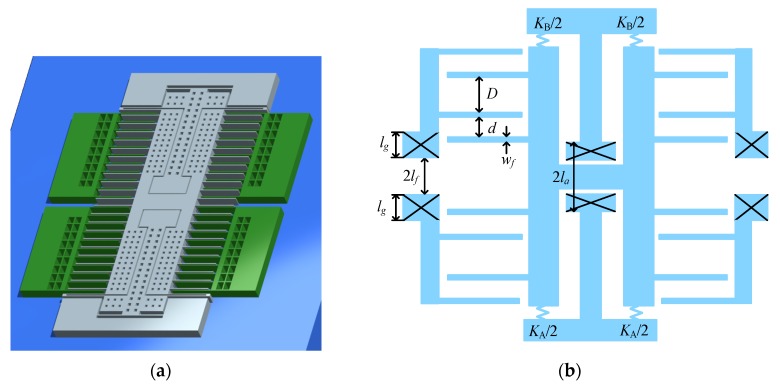
The improved structure for MEMS accelerometer. (**a**) Overall layout; (**b**) Scheme diagram of dimensions for the comb capacitor.

In this section, the ability of an improved structure (shown in Figure 4) that can avoid the contrary effect is illustrated. The improved structure is proposed in our previous work for achieving low TCB and TCSF. For the improved structure, TCSF is expressed as [6].

(10) $$TCSF=TCSF_{STD}+TCSF_{TS}$$

(11) $$TCSF_{STD}=-TCS$$

(12) $$TCSF_{TS}=-\alpha_{s}-\frac{\eta^{2}+1}{\eta^{2}-\eta }\frac{l_{f}+l_{g}}{d}\left(\alpha_{eq}-\alpha_{s}\right)$$

where *l_g_* is the length of anchors for fixed electrodes, TCSF_STD_ and TCSF_TS_ denote the TCSF induced by the temperature dependence of the spring stiffness and thermal stress, respectively.

The improved structure does not change the detecting principle and electrode layout, so the linearity error is still expressed by Equation (8). In this work, two coefficients are defined to represent the effect of WNGR on linearity error and TCSF

(13) $$\begin{array}{l} \beta_{l\_error}=\frac{1}{\eta }\\ \beta_{TCSF}=\frac{\eta^{2}+1}{\eta^{2}-\eta } \end{array}$$

**Figure 5:**
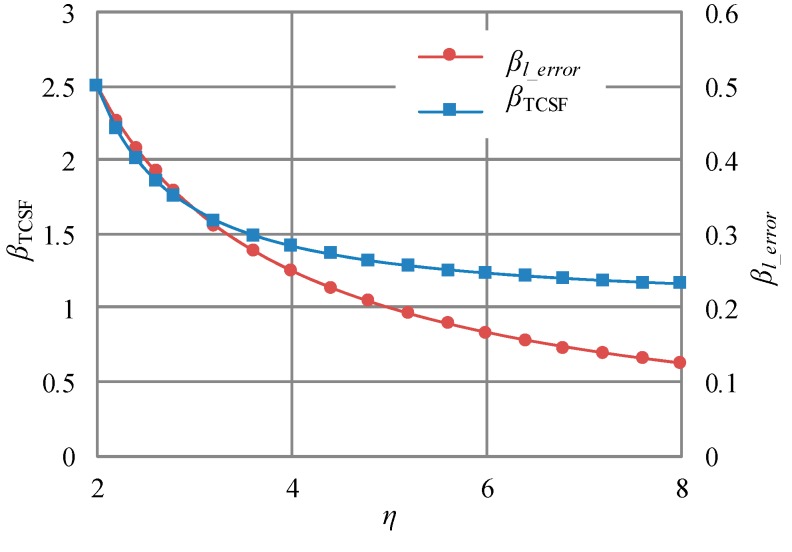
The effect of WNGR on *β_l_error_* and *β*_TCSF_.

According to Equations (8) and (10), the linearity error decreases with decreasing *β_l_error_*, and TCSF decreases with decreasing *β*_TCSF_. The relationship between *β_l_error_* and WNGR is shown in Figure 5, and so is the relationship between *β*_TCSF_ and WNGR. It can be seen that *β_l_error_* and *β*_TCSF_ both decrease with increasing WNGR. As such, the improved structure avoids the contrary effect of WNGR on TCSF and linearity error.

Then, as long as precise parameters are employed, the very low temperature coefficient and linearity error can both be obtained with the improved structure. In the next section, a precise design for the improved structure is developed.

## 4. Precise Design for both Low Temperature Coefficient and Linearity Error

### 4.1. Design for Low TCSF and Linearity Error

From Equation (10), it is known that TCSF consists of TCSF_STD_ and TCSF_TS_. TCSF_STD_ and TCSF_TS_ cancel each other. TCSF_STD_ is equal to the opposite of TCS. TCS is about −30 ppm/°C, as explained in Section 3.2. Thus, if TCSF_TS_ is −30 ppm/°C, TCSF is null. However, the absolute value of TCSF_TS_ is much higher than 30 ppm/°C [6]. As such, TCSF_TS_ must be reduced. TCSF_TS_ is directly proportional to the CTE difference (*α_eq_* − *α_s_*). According to the study in Section 3.2, *α_eq_* is higher than *α_s_*_._ Thus, TCSF_TS_ decreases with decreasing *α_eq_*. The minimum *α_eq_* is about 3.25 ppm/°C and is achieved by the soft die-attach. Therefore, the minimum *α_eq_* is employed in this work.

**Figure 6:**
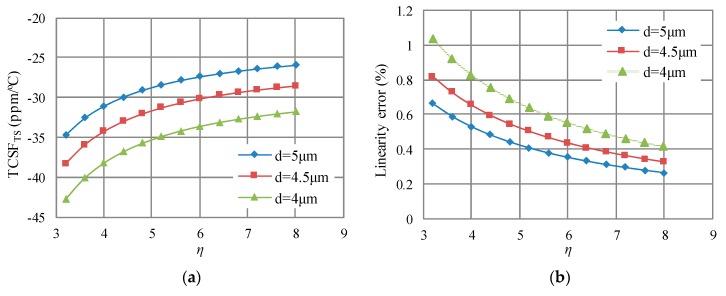
The effect of the narrow gap (*d*) and WNGR (*η*) on the TCSF_TS_ and linearity error. (**a**) TCSF_TS_; (**b**) Linearity error.

Besides, TCSF_TS_ also varies with the narrow gap and WNGR. The effect of the narrow gap and WNGR on TCSF_TS_ and linearity error is shown in Figure 6. The length of anchors for fixed electrodes *l_g_* is 110 μm, and other parameters are listed in Table 1. These employed parameters are the same as those in the previous work except the *α_eq_*.

For the narrow gap of 5 μm employed in the previous work, it can be seen that WNGR should be 4.4 to make TCSF_TS_ close to −30 ppm/°C. On the other side, with the narrow gap of 4.5 μm and WNGR of 6.5, a TCSF_TS_ close to −30 ppm/°C can also be achieved. With the two sets of the narrow gap and WNGR, both low linearity errors can be achieved, and they are about 0.42% and 0.4%, respectively. Because lower narrow gap results in higher capacitances, the narrow gap of 4.5 μm and WNGR of 6.5 are selected in this work.

Another important problem is the increased length of the comb capacitor induced by the dimension variation. From Figure 4, the length of the comb capacitor is expressed as

(14) $$L_{comb}=\left(N-1\right)\left(d+d\eta +2w_{f}\right)+w_{f}$$

For the narrow gap of 5 μm and WNGR of 5 employed in the previous work, *L_comb_* is 866.5 μm. On the other side, for the narrow gap of 4.5 μm and WNGR of 6.5, *L_comb_* is 941.5 μm. The increasing of *L_comb_* is very small compared to the overall size of the die (3200 μm × 3200 μm). As such, the increasing of *L_comb_* does not result in a huge impact on the die yield. 

### 4.2. Designing for Low TCB 

Besides TCSF, TCB is another important parameter affecting the temperature performance of MEMS accelerometers. According to the previous work [6], TCB is expressed as

(15) $$\mathrm{TCB}=\frac{K_{A}-K_{B}}{m}\left(\alpha_{eq}-\alpha_{s}\right)l_{a}$$

where *l_a_* is the distance from the anchor for movable electrodes to the midline as depicted in Figure 4, *K_A_* and *K_B_* denote the stiffness of the springs connecting proof mass.

**Figure 7:**
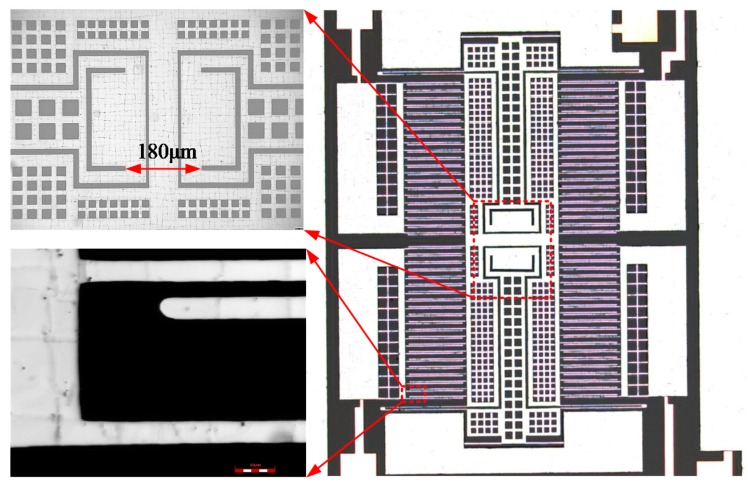
The microscope image of the accelerometer.

TCB also decreases with decreasing equivalent CTE. The minimum *α_eq_* is equal to the CTE of the substrate. If the silicon substrate is employed, the minimum *α_eq_* is equal to the CTE of silicon. As a result, TCB disappears. TCSF_TS_ also disappears. However, TCSF can’t be close to zero because TCSF_STD_ still exists. In this work, the glass substrate is employed to achieve null TCSF by making TCSF_TS_ and TCSF_STD_ cancel each other. TCB is reduced by other methods, such as shorter *l_a_*. In this work, the anchor for movable electrodes is redesigned, as depicted in Figure 7. Through the redesigning of the anchor, *l_a_* is reduced from 190 μm (employed in the previous work) to 90 μm. Changing the stiffness or mass may also reduce TCB. However, this results in the variation of linearity error. As such, the spring stiffness and mass remain the same as those in the previous work.

Because the stiffness difference (*K_A_* − *K_B_*) is induced by random fabrication errors [6], it is difficult to estimate the accurate TCB. However, the improvement of TCB can be estimated based on Equation (15). The CTE difference (*α_eq_* − *α_s_*) is about 0.65 ppm/°C in this work, while that in the previous work is about 1.6 ppm/°C [6]. As a result, the estimated TCB in this work is about 20% of that in the previous work.

Integrating the designing works on the TCSF, TCB, and linearity error, the modifications on the improved structure are summarized as follows.

(1) In order to achieve the minimum *α_eq_*, a soft adhesive is employed for the die-attach.
(2) The narrow gap *d* is decreased from 5 μm to 4.5 μm.
(3) In order to make WNGR be 6.5, the wide gap *D* is modified to be 29.3 μm.
(4) The distance from the anchor for moving electrodes to midline *l_a_* is decreased from 190 μm to 90 μm.

The other designing parameters are the same as those employed in the previous work. The parameter and performance differences made by these modifications between this work and the previous work are listed in Table 2.

## 5. Experiments

### 5.1. Temperature Coefficients

**Figure 8:**
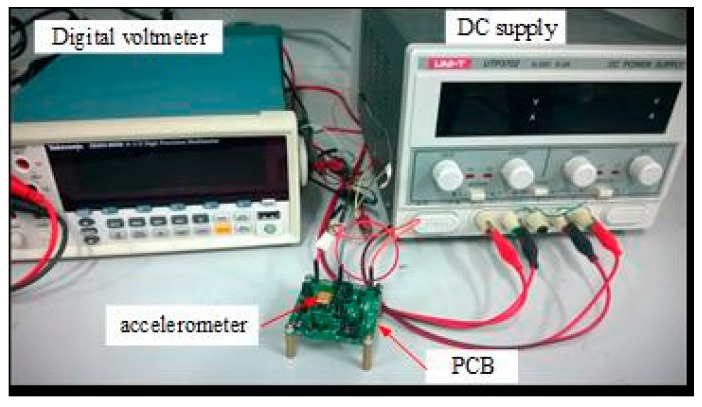
Testing equipment for accelerometers. DC supply and digital voltmeter provide the power input and display for output voltage, respectively.

In this work, accelerometers with the modified dimensions were fabricated. The microscope image of the accelerometer is shown in Figure 7. In order to reduce TCSF and TCB, the accelerometer die is attached to the ceramic package by a soft adhesive with Young's modulus lower than 10 MPa. The input, output, and ground pads are wire-bonded to the copper trace on the ceramic package. The testing principle of the self-balancing bridge is implemented in the printed circuit board (PCB) using the discrete components. The packaged accelerometer is mounted on the PCB for testing, as shown in Figure 8. 

In order to measure TCSF and TCB, the scale factor and bias under different temperatures must be measured. The measuring method is detailed before in [6]. With the measuring results of the scale factor and bias under different temperatures, TCSF and TCB are calculated by the following equations:

(16) $$TCSF=\frac{k_{1}\left(T+\Delta T\right)-k_{1}\left(T-\Delta T\right)}{2\Delta Tk_{1}\left(T\right)}$$

(17) $$TCB=\frac{p_{0}\left(T+\Delta T\right)-p_{0}\left(T-\Delta T\right)}{2\Delta T}$$

where *p*_0_ and *k*_1_ denote the bias and scale factor, respectively. In this work, the scale factor and bias under 5 °C, 15 °C, 25 °C, 35 °C, 45 °C, 55 °C, 65 °C, 75 °C, and 85 °C was measured, so TCSF and TCB under 15 °C, 25 °C, 35 °C, 45 °C, 55 °C, 65 °C, and 75 °C are obtained.

**Figure 9:**
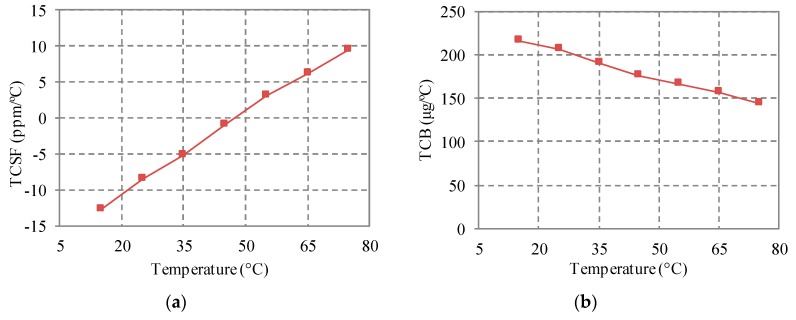
Temperature dependences of TCSF and TCB. (**a**) TCSF; (**b**) TCB.

The temperature dependences of TCSF and TCB are shown in Figure 9. TCB decreases with the temperature and the relationship is approximately linear. This temperature dependence may be induced by the temperature dependence of the CTE of silicon. According to Equation (15), it is known that TCB has a linear relationship with the CTE difference (*α_eq_* − *α_s_*). *α_eq_* is equal to CTE of the Pyrex 7740 glass. The result in the literature [20] shows that CTE of the Pyrex 7740 glass is almost constant from 0 °C to 100 °C. CTE of silicon increases almost linearly from 0 °C to 100 °C, but is still lower than CTE of the Pyrex 7740 glass [21]. As a result, the CTE difference (*α_eq_* − *α_s_*) decreases with increasing temperature linearly. As such, TCB decreases with increasing temperature.

The relationship between TCSF and temperature is also approximately linear. However, TCSF increases with increasing temperature. From Equation (10), the temperature dependence of TCSF can be induced by temperature dependences of (*α_eq_* − *α_s_*) and TCS. However, TCS is almost constant from 0 °C to 100 °C [19]. As such, the approximately linear relationship between TCSF and temperature is also caused by the temperature dependence of the CTE of silicon.

**Figure 10:**
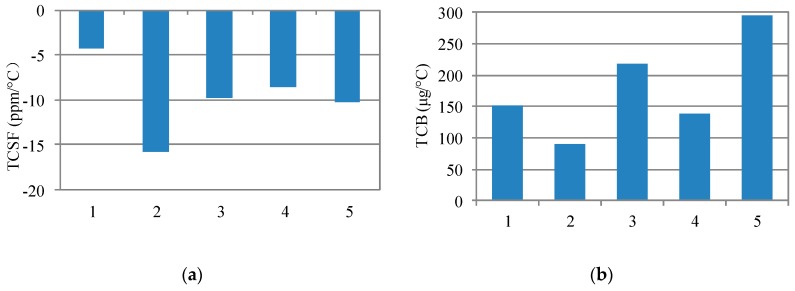
Measured results of TCSF and TCB for five accelerometers under room temperature (25 °C). (**a**) TCSF; (**b**) TCB.

More results of TCSF and TCB under room temperature (25 °C) are shown in Figure 10. The five results of TCSF are all very low. The max TCSF is −16.1 ppm/°C, and the average TCSF is about −9.8 ppm/°C. The significant improvement on TCSF is also verified by the comparison in Table 3. The max TCB is 294 μg/°C, and the average TCB is 179 μg/°C. TCB is in the same order of those found in the literature [4,13,14,15,16]. The comparison in Table 3 also verifies the significant improvement on TCB.

Though TCSF and TCB are both reduced, there exists deviation between the experimental results and the theoretical results estimated in Section 4. For example, the average TCSF of −9.8 ppm/°C deviates from the theoretical result that is close to zero. The average of the measured TCB is about 34.4% of that in the previous work. However, the estimation shows that the theoretical TCB estimated in this work is about 20% of that estimated in the previous work. These deviations indicate that there exist some other factors affecting TCSF and TCB, such as the temperature dependence of the circuit. Therefore, more factors must be considered in the future study.

### 5.2. Linearity Error

The linearity error is measured by precision centrifuge testing for linear accelerometers. In short, an accelerometer is mounted on the centrifuge, and the sensitive direction is along with the radius. Then, the accelerometer is tested under different centrifugal accelerations. The distribution of centrifugal accelerations is [0 g, 5 g, 10 g, 15 g, 20 g, 15 g, 10 g, 5 g, 0 g]. Then, the accelerometer is remounted to invert the sensitive direction, so the output under the negative accelerations are tested. When the output under different accelerations is obtained, a least-squares linear fit of the input-output data is made. Finally, the linearity error is calculated by Equation (7).

**Figure 11:**
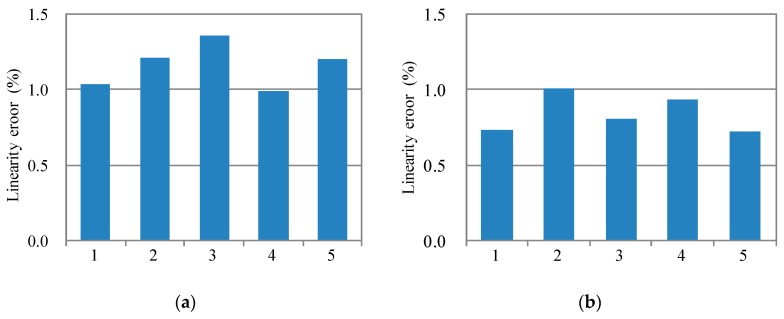
Comparison of the experimental results of linearity error. (**a**) Experimental results of linearity error of the accelerometers designed in this work; (**b**) Experimental results of linearity error of the accelerometers designed in the previous work.

The measuring results of linearity error are shown in Figure 11. The average linearity error for the five accelerometers designed in this work is about 0.84%. On the other side, the average linearity error for five accelerometers designed in the previous work is about 1.16%. The measured linearity error in this work is 72% of that in the previous work. As such, the lower linearity error is achieved through the increasing of WNGR. 

However, there exist deviations between the measured results and the theoretical results. Firstly, the measured linearity error of 0.84% is higher than the theoretical linearity error of 0.4% listed in Table 2. Secondly, the result in Table 2 shows that the estimated linearity error for the accelerometers in this work is slightly lower than that in the previous work. These deviations may be made by the fringe capacitance. The results in the literature [22] show that the fringe capacitance also influences the linearity error. Because the linearity error model established in this work does not consider the fringe capacitance, the estimated linearity error may underestimate the actual linearity error. In addition, the decreased narrow gap decreases the proportion of fringe capacitance in the total capacitance. As a result, the influence of fringe capacitance on the linearity error is decreased. In other words, the linearity error is decreased by the decreasing of the narrow gap. As a result, the theoretical estimated result underestimates the actual improvement in the linearity error.

## 6. Conclusions

In this work, a structural design for a bulk MEMS capacitive accelerometer is proposed for both low temperature coefficient and linearity error. Firstly, the contrary effect of WNGR on TCSF and linearity error is illustrated and is avoided by an improved structure. Within the improved structure, both TCSF and the linearity error decrease with increasing WNGR. Then, the precise design of the improved structure is developed for achieving much lower TCB and TCSF with the precondition of high linearity. Finally, the structural design for low TCB, TCSF and linearity error is experimentally verified. Experimental results show that the new structural design results in a linearity error close to 0.84%, a TCB close to 179 μg/°C, and a TCSF close to −9.8 ppm/°C.

In future, more imperfect factors, such as the fringe capacitance and the temperature dependence of the circuit, need to be coupled into the models for temperature coefficients and linearity error to enable a more precise design. In addition, more parameters could be integrated into research, such as mechanical noise, bias, and so on.

## Figures and Tables

**Table 1:** Parameters for the MEMS accelerometer.

Parameter	Value	Units	Parameter	Value	Units
Mass (*m*)	59.4	μg	Electrode width (*w_f_*)	6.5	μm
Fixed electrodes number in a comb structure (*N*)	21		Distance from the first fixed electrode to midline (*l_f_*)	45	μm
Stiffness of springs (*K*)	16	N/m	Equivalent CTE (*α_eq_*)	3.25	ppm/°C
maximum acceleration (*a*_max_)	20	g	Silicon CTE (*α_s_*) [6]	2.6	ppm/°C
Narrow gap (*d*)	5	μm	*TCS* [6]	−30	ppm/°C
WNGR (*η*)	5				

**Table 2:** The parameter and performance differences between this work and the previous work.

Parameter	In This Work	In Previous Work	Units
Narrow gap (*d*)	4.5	5	μm
Wide gap (*D*)	29.3	25	μm
Distance from anchors for moving electrodes to midline (*l_a_*)	90	190	μm
Equivalent CTE (*α_eq_*)	3.25	4.2	ppm/°C
Linearity error	0.4%	0.42%	
TCSF	almost zero	37	ppm/°C
TCB	The estimated TCB in this work is about 20% of that in the previous work		

**Table 3:** Comparison of measured TCSF and TCB between this work and the previous work.

Parameter	In This Work	In Previous Work	Units
TCSF	average: −9.8	average: −50.8	ppm/°C
	max: −16.1	max: −62.6	
TCB	average: 179	average: 520	μg/°C
	max: 294	max: 1033	
